# The Trans-Achilles Approach for Plate Supplementation of Ankle Arthrodesis With an Existing Hindfoot Fusion Nail: A Case Report

**DOI:** 10.7759/cureus.39569

**Published:** 2023-05-27

**Authors:** Ahmed H Elhessy, Abhijith Annasamudram, Stephanie Wu, Janet D Conway

**Affiliations:** 1 Medicine, University of Maryland School of Medicine, Baltimore, USA; 2 Orthopedics, The Rubin Institute for Advanced Orthopedics/Sinai Hospital, Baltimore, USA; 3 Podiatry, International Center for Limb Lengthening, Rubin Institute for Advanced Orthopedics, Baltimore, USA; 4 Orthopedics, International Center for Limb Lengthening, Rubin Institute for Advanced Orthopedics, Baltimore, USA

**Keywords:** approach, trans-achilles, nonunion, hindfoot fusion, ankle arthrodesis

## Abstract

Tibiotalar arthrodesis revision surgeries are not uncommon. Several approaches have been described in the literature for ankle arthrodesis nonunions. In this article, we describe the posterior trans-Achilles approach, which ensures adequate surgical exposure while minimizing damage to the surrounding soft tissues. It provides a convenient method for utilizing bone grafts or substitutes and allows for the advantageous application of posterior plating. The possible complications of this approach are delayed wound healing, wound infection, injury to the sural nerve, and the potential need for a skin graft. Despite the advantages of this approach, infection, delayed union, and nonunion risks remain high in this patient population. Finally, the trans-Achilles approach is valid for complex ankle procedures, especially in revisions with compromised ankle soft tissue envelopes.

## Introduction

Despite the advances in modern implants and surgical techniques, tibiotalar arthrodesis remains commonly associated with postoperative nonunion and leads to revision procedures [1,2]. Nonunion risk factors are multifactorial, with smoking being the most common risk factor [3]. The surgical approach for any hindfoot arthrodesis should be cautiously considered in order to prevent additional damage to soft tissues that are already compromised. Typical approaches for such fusions include the transfibular, modified medial, posterolateral, and posterior techniques [4]. We describe the posterior trans-Achilles approach for ankle arthrodesis nonunion revisions. This approach provides the advantage of reduced soft tissue damage, improved exposure, and posterior plating techniques [4,5].

This report is based upon a case series of 10 patients with recalcitrant ankle nonunion treated with a combination of a hindfoot arthrodesis rod and supplemental posterior plate fixation.

## Case presentation

**Figure 1:**
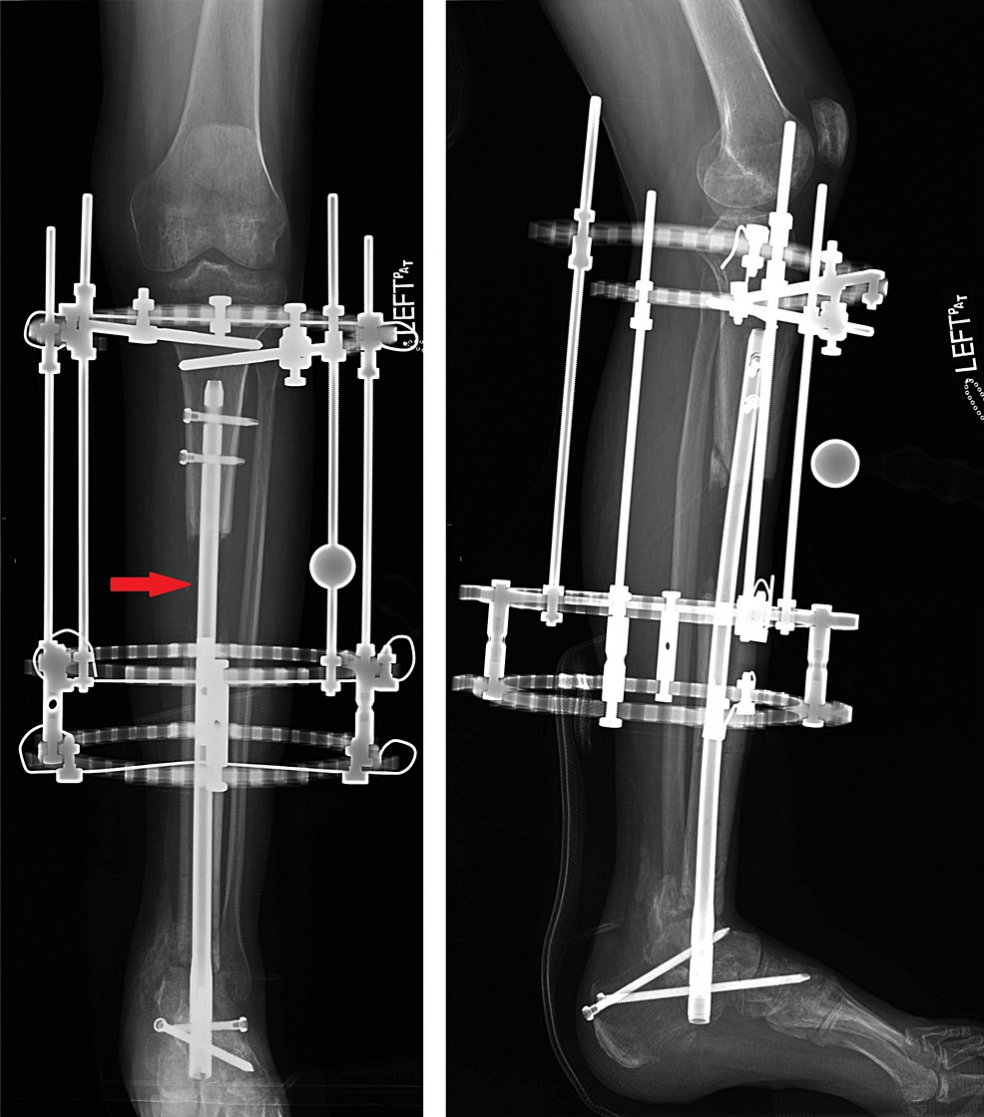
Pre-operative plain radiographs of the left leg show an Intramedullary nail and external fixator showing the tibial segmental bone loss (red arrow) and ankle fusion nonunion.

We present a case of a 42-year-old male who sustained an infected left pilon fracture after a motor vehicle accident. The injury was initially treated with open reduction and internal fixation. During the follow-up, the patient developed left tibia chronic osteomyelitis. Due to the complexity of the case, it was treated with an 8 cm segmental tibial resection and bone transport using a circular frame (transporting in a proximal to distal direction) over a long antibiotic-coated ankle arthrodesis nail (Figure 1).

The patient's surgical scars were on both the anterior and lateral aspects. These incisions were used for segmental tibial resection, tarsal tunnel release, and ankle fusion. The bone transport was set at a rate of 0.25 mm four times a day.

After three months of the salvage procedure, the patient was taken to the operating room under general anesthesia. Then, bone graft harvesting from the femur using the reamer-irrigator-aspirator (RIA) was performed before the trans-Achilles approach.

Bone graft harvesting technique

The external fixator and lower limb were prepared, draped into the sterile field, and covered during the femur's retrograde intramedullary bone graft harvesting. A Steinmann pin and anterior cruciate ligament (ACL) reamer were used to access the intramedullary canal of the femur in a retrograde fashion. This was confirmed using C-arm fluoroscopy. Next, a 1 cm trans-patellar tendon incision was utilized to access the ipsilateral distal femur for bone graft harvesting via the RIA. A 2.5 mm guide rod was introduced and checked under image intensifier guidance. Forty cc of bone graft material was collected from the distal femur. The 1 cm incision was then reapproximated with staples and polydioxanone suture (PDS) stitches. Bone graft harvest using the RIA technique is proven to provide an average volume of 50 ml of bone graft [6].

Trans-Achilles approach

**Figure 2:**
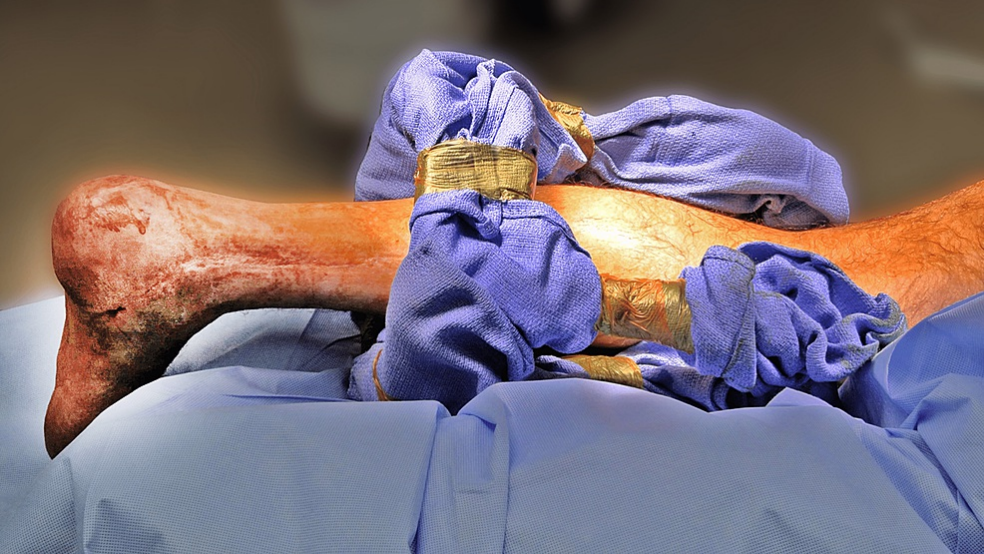
Intraoperative clinical image showing the preparation and positioning of the leg

After standard sterile draping, two sterile bumps were placed underneath the knee joint with soft padding to all pressure points on the operating room table. Although we did not perform this surgery under a tourniquet, the tourniquet is to be applied according to the operating surgeon’s preference (Figure 2).

**Figure 3:**
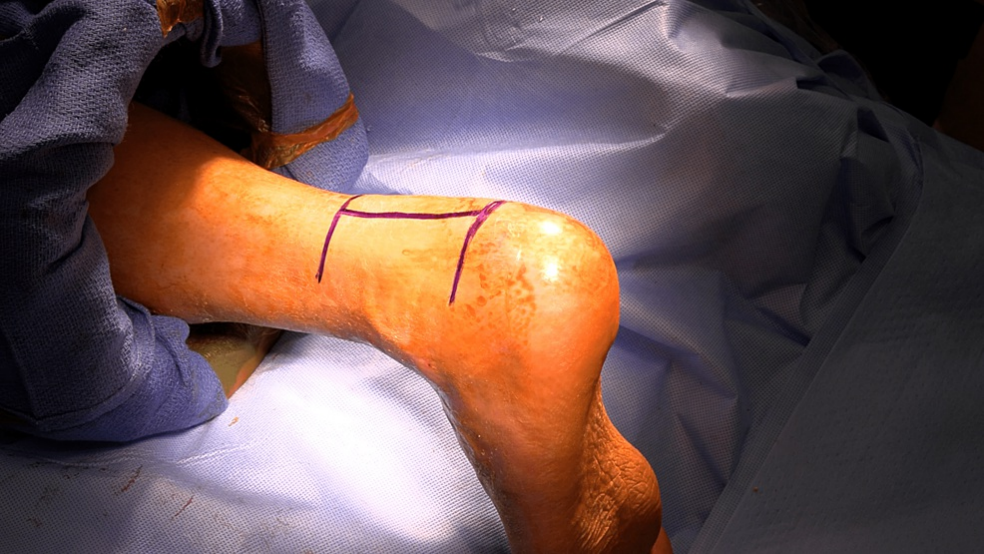
Intraoperative image showing the marking of the surgical incision

Under fluoroscopy guidance, a midline longitudinal skin incision was made with a scalpel 5 cm above and 5 cm below the ankle joint (Figure 3).

**Figure 4:**
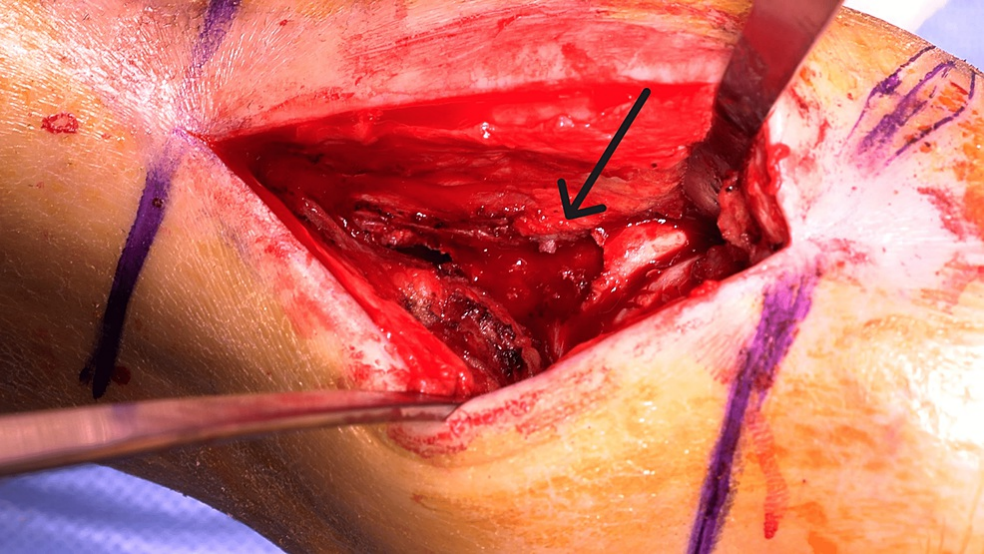
Intraoperative image showing good exposure of the nonunion site (arrow) between the distal tibia and the talus.

Next, a sharp dissection was performed distally through the skin and the Achilles tendon into the posterior fat pad to prevent potential wound-healing complications. The flexor hallucis longus (FHL) tendon and neurovascular bundle were identified and retracted away from the posterior aspect of the tibia, allowing maximum exposure of the operative field. Gentle retraction at the skin edges aids in minimizing the risk of skin necrosis related to excessive pressure. The authors recommend using an Army-Navy (or similar) retractor instead of a self-retainer to reduce wound healing problems. A Bovie was used for hemostasis and dissection to the osseous layer. The nonunion is well-visualized intraoperatively with the surgical exposure the trans-Achilles approach offers (Figure 4).

Next, the fusion site was prepared using a bone burr to obtain clean bleeding bone edges. At this point, the autologous bone graft for the ankle joint previously harvested from the ipsilateral distal femur was packed down with a Cobb elevator using a clean lap sponge to absorb any excess fluid. This step was repeated several times until enough bone graft filled the entire defect. It was then soaked in a bone morphogenetic protein-2 (BMP-2) graft and placed on the defective or nonunion site. Fluoroscopy guidance is essential to identify further bone defects requiring additional bone grafts.

An eight-holed posterior plate was contoured and fixed using 3.5 mm screws. Three screws each were used proximally and distally to secure the plate. The ankle was maintained in neutral flexion/extension and 10 degrees of external rotation. One gram of vancomycin powder was applied to the site, and then the incision was reapproximated in a layered fashion for primary wound closure. Postoperatively, the extremity was well-padded with a posterior splint.

**Figure 5:**
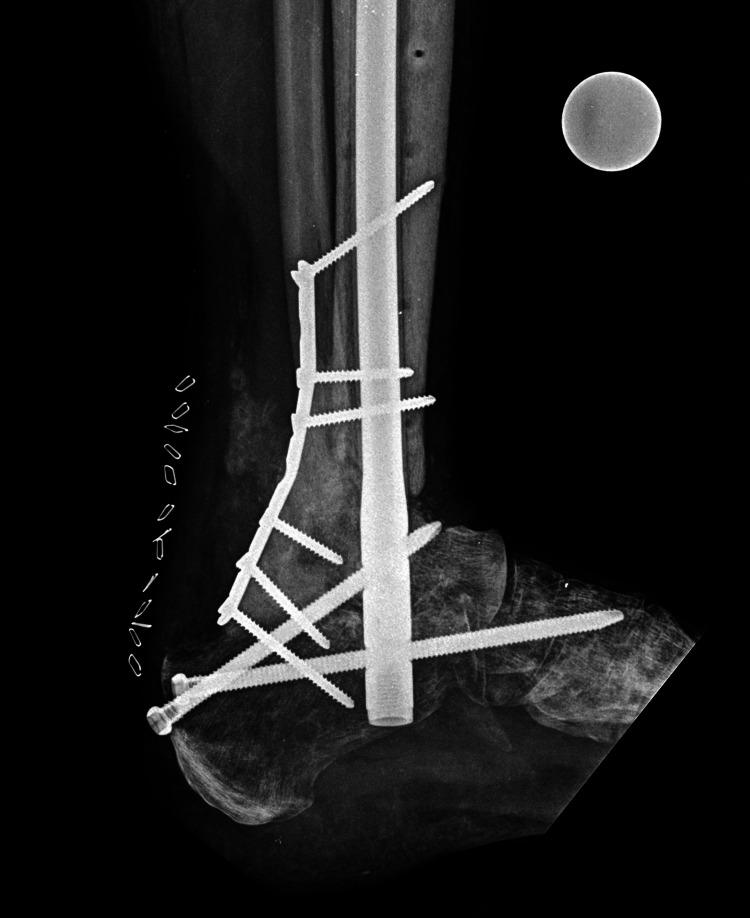
Lateral plain radiograph showing bone bridging and healed ankle fusion

**Figure 6:**
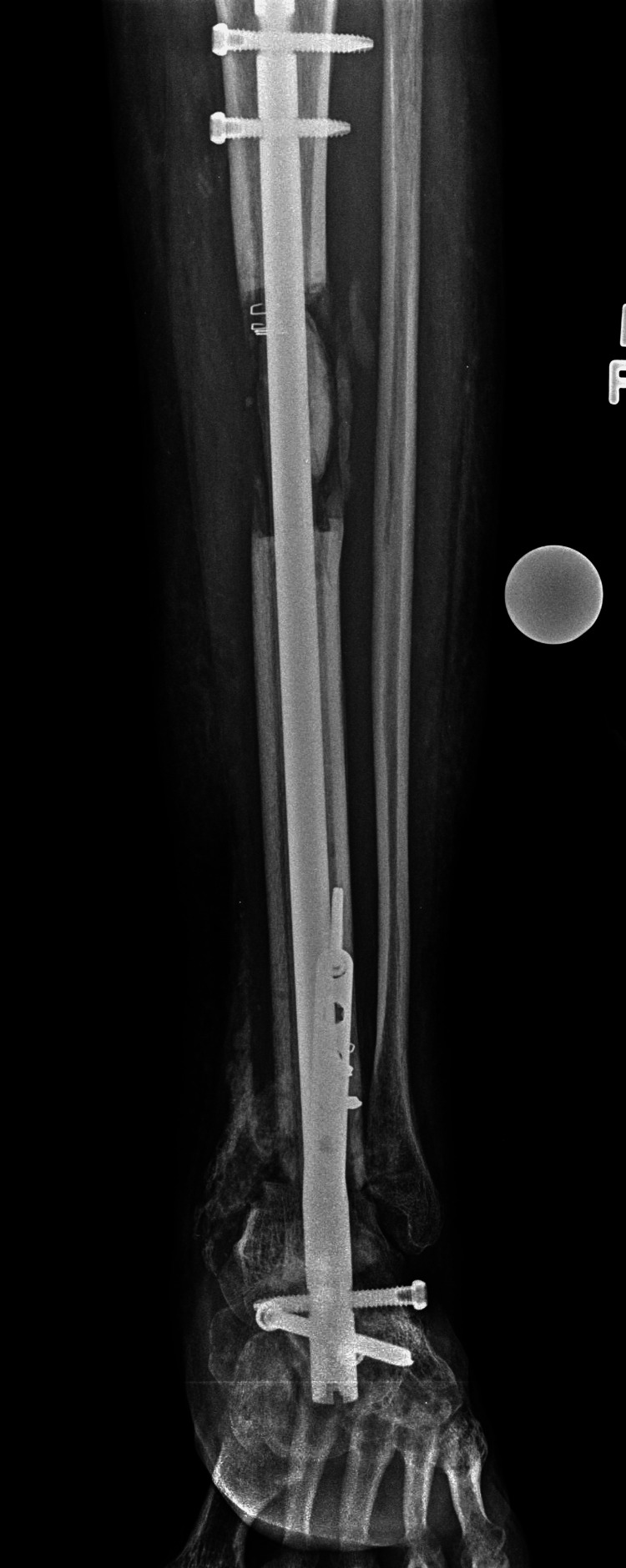
AP plain radiograph showing bone regeneration (proximally) and healed ankle fusion

The postoperative protocol included; six weeks of non-weight-bearing using a Charcot restraint orthotic walker (CROW) boot, after which the patient was allowed to weight-bear as tolerated. The ankle arthrodesis showed clinical and radiological signs of bone healing after five months after the procedure (Figures 5, 6).

## Discussion

The trans-Achilles approach is reproducible, preserves angiosomes, and provides wide exposure for hardware placement, which can provide biomechanical stability [7-9]. In addition, this approach aids in the retention of harvested bone graft material, thus assisting in bone healing in malunited or nonunited ankle fusions. Also, the iliac crest bone graft remains a feasible option with the prone position [10].

In a retrospective review, Pellegrini et al. reported the outcomes of 41 patients who underwent tibiotalocalcaneal arthrodesis via a posterior Achilles tendon splitting approach for several reasons, including nonunion. Although the fusion rate was more than 80%, complications were reported in more than 41% of their series. Ankle nonunion remained the most commonly reported complication, and one patient ended with an amputation. Their results support the complexity of treating such conditions [5].

Using self-retaining retractors in surgery can have potential complications; these complications can be related to improper placement or excessive retraction force that can lead to tissue trauma. Also, prolonged and excessive pressure on the tissues due to the retractor blades can restrict the blood flow to the area, potentially causing ischemia and tissue necrosis [11]. We encourage surgeons to exercise caution, proper technique, and continuous monitoring while using self-retaining retractors to minimize the occurrence of complications that might be related to excessive soft tissue retraction.

Additionally, this approach provides adequate hindfoot exposure for open reduction and plate fixation [9]. Furthermore, the trans-Achilles approach avoids a fibular osteotomy (that occurs when using the lateral approach instead), which could create an unnecessary postoperative valgus tilt as a complication [12]. Finally, keeping the fibula intact without disruption will allow for potential total ankle arthroplasty in the future should it be deemed necessary [5,13]. Although a tourniquet can be used for ankle procedures, we prefer to avoid using a tourniquet, provided that we can preserve adequate visualization via a traumatic dissection in layers and utilization of electrocautery throughout the procedure [14]. Also, we prefer to apply vancomycin powder prior to skin closure in diabetic patients and complex revision cases. Applying 500 mg to 1000 mg of local vancomycin powder is proven to reduce the surgical site infection rate in diabetic patients undergoing foot and ankle surgeries [15].

Complications are usually related to the reduced blood supply in this area in addition to the patient’s age, comorbidities, and soft tissue condition related to previous interventions. The possible complications of this approach are delayed wound healing, wound infection, neurovascular injury, and the potential need for a skin graft. Despite the advantages of the approach, augmentation with bone grafting, bone substitutes, and rigid fixation, these patients are still susceptible to the risk of infection, delayed union, or nonunion. This could be related to the diminished blood supply, advancing age, medical comorbidities, and recurrent surgical interventions, which can compromise the soft tissue [16]. There is also a chance of the posterior aspect of the skin breakdown resulting in plate exposure. Hence, a well-padded posterior aspect of the distal leg and foot postoperatively is needed to avert this dreadful complication.

## Conclusions

Ankle arthrodesis nonunions are challenging to any surgeon for many reasons, including the complexity of the pathology, the patient's general condition, associated comorbidities, local soft tissue conditions, vascularity, infection, and bone loss. The trans-Achilles approach represents an approach that can minimize soft tissue compromise, provide wide exposure for complex reconstruction, and preserve bone integrity.
